# Supplementary material for: A deletion polymorphism in the Caenorhabditis elegans RIG-I homolog disables viral RNA dicing and antiviral immunity
Source: eLife. 2013 Oct 8;2:e00994. doi: 10.7554/eLife.00994 (PMC3793227; doi:10.7554/eLife.00994)
Supplement: Supplementary file 1. — DOI: http://dx.doi.org/10.7554/eLife.00994.020 [file elife00994s001.docx]

**Supplementary File 1**

**A**

**Table 1: Strains used in this study**

| **Strain** | **Description** | **Haplotype in the *drh-1* region** |
| --- | --- | --- |
| AB1 | Natural isolate from Australia. | AB1 |
| AB4 | Natural isolate from Australia. | AB4 |
| CB4854 | Natural isolate from United States of America. | PX179 |
| CB4856 | Natural isolate from Hawaii. | CB4856 |
| CB4857 | Natural isolate from United States of America. | PS2025 |
| CB4858 | Natural isolate from United States of America. | PS2025 |
| CB4932 | Natural isolate from United Kingdom. | JU1580 |
| CX11262 | Natural isolate from United States of America. | PS2025 |
| CX11264 | Natural isolate from United States of America. | PS2025 |
| CX11271 | Natural isolate from United States of America. | PS2025 |
| CX11276 | Natural isolate from United States of America. | PS2025 |
| CX11285 | Natural isolate from United States of America. | N2 |
| CX11292 | Natural isolate from United States of America. | PS2025 |
| CX11307 | Natural isolate from United States of America. | N2 |
| CX11314 | Natural isolate from United States of America. | PS2025 |
| CX11315 | Natural isolate from United States of America. | PS2025 |
| DL200 | Natural isolate from Ethiopia. | N2 |
| DL226 | Natural isolate from United States of America. | N2 |
| DL238 | Natural isolate from United States of America. | DL238 |
| DR1350 | Natural isolate from Hawaii. | PS2025 |
| ED3005 | Natural isolate from United Kingdom. | PS2025 |
| ED3010 | Natural isolate from United Kingdom. | JU1200 |
| ED3011 | Natural isolate from United Kingdom. | PS2025 |
| ED3017 | Natural isolate from United Kingdom. | N2 |
| ED3040 | Natural isolate from South Africa. | N2 |
| ED3046 | Natural isolate from South Africa. | N2 |
| ED3048 | Natural isolate from South Africa. | N2 |
| ED3049 | Natural isolate from South Africa. | N2 |
| ED3052 | Natural isolate from South Africa. | JU1580 |
| ED3073 | Natural isolate from Kenya. | N2 |
| ED3077 | Natural isolate from Kenya. | N2 |
| EG4346 | Natural isolate from United States of America. | PS2025 |
| EG4349 | Natural isolate from United States of America. | EG4349 |
| EG4724 | Natural isolate from Portugal. | EG4724 |
| EG4725 | Natural isolate from Portugal. | EG4724 |
| EG4946 | Natural isolate from United States of America. | PS2025 |
| JT11398 | Natural isolate from United States of America. | N2 |
| JU1088 | Natural isolate from Japan. | N2 |
| JU1171 | Natural isolate from Chile. | JU1171 |
| JU1172 | Natural isolate from Chile. | PS2025 |
| JU1200 | Natural isolate from United Kingdom. | JU1200 |
| JU1212 | Natural isolate from France. | JU1580 |
| JU1213 | Natural isolate from France. | JU1580 |
| JU1242 | Natural isolate from France. | JU1242 |
| JU1246 | Natural isolate from France. | JU1580 |
| JU1395 | Natural isolate from France. | N2 |
| JU1400 | Natural isolate from Spain. | N2 |
| JU1401 | Natural isolate from Spain. | JU1401 |
| JU1440 | Natural isolate from Spain. | JU1401 |
| JU1491 | Natural isolate from France. | JU1580 |
| JU1530 | Natural isolate from France. | N2 |
| JU1563 | Natural isolate from France. | N2 |
| JU1568 | Natural isolate from France. | N2 |
| JU1580 | Natural isolate from France. Originally infected with the Orsay virus. | JU1580 |
| JU1581 | Natural isolate from France. | JU1580 |
| JU1586 | Natural isolate from France. | N2 |
| JU1652 | Natural isolate from Uruguay. | RW7000 |
| JU1896 | Natural isolate from Greece. | RW7000 |
| JU258 | Natural isolate from Madeira. | JU258 |
| JU310 | Natural isolate from France. | N2 |
| JU311 | Natural isolate from France. | JU311 |
| JU323 | Natural isolate from France. | JU323 |
| JU346 | Natural isolate from France. | JU346 |
| JU363 | Natural isolate from France. | N2 |
| JU367 | Natural isolate from France. | N2 |
| JU393 | Natural isolate from France. | JU393 |
| JU394 | Natural isolate from France. | JU394 |
| JU397 | Natural isolate from France. | JU1580 |
| JU406 | Natural isolate from France. | JU1580 |
| JU440 | Natural isolate from France. | N2 |
| JU561 | Natural isolate from France. | JU561 |
| JU642 | Natural isolate from France. | N2 |
| JU751 | Natural isolate from France. | EG4724 |
| JU774 | Natural isolate from Portugal. | N2 |
| JU775 | Natural isolate from Portugal. | JU775 |
| JU778 | Natural isolate from Portugal. | JU778 |
| JU782 | Natural isolate from Portugal. | JU782 |
| JU792 | Natural isolate from France. | JU1580 |
| JU801 | Natural isolate from France. | N2 |
| JU830 | Natural isolate from Germany. | N2 |
| JU847 | Natural isolate from France. | JU1580 |
| KR314 | Natural isolate from Canada. | N2 |
| LKC34 | Natural isolate from Madagascar. | JU1580 |
| MY1 | Natural isolate from Germany. | N2 |
| MY10 | Natural isolate from Germany. | MY10 |
| MY16 | Natural isolate from Germany. | JU1171 |
| MY18 | Natural isolate from Germany. | PS2025 |
| N2 | Standard laboratory strain. Initially isolated in the United Kingdom. | N2 |
| PB303 | Natural isolate from United States of America. | N2 |
| PB306 | Natural isolate from United States of America. | PS2025 |
| PS2025 | Natural isolate from United States of America. | PS2025 |
| PX179 | Natural isolate from United States of America. | PX179 |
| QX1211 | Natural isolate from United States of America. | QX1211 |
| QX1233 | Natural isolate from United States of America. | QX1233 |
| RC301 | Natural isolate from Germany. | N2 |
| RW7000 | Natural isolate from France. | RW7000 |
| WN2002 | Natural isolate from the Netherlands. | N2 |
| JU2170 | *mfIR38(IV,JU1580>N2);*  IV:3329219 to IV:11083410 is JU1580; IV:2279105 and IV:15829314 are N2. |  |
| JU2194 | *mfIR39 (IV,JU1580>N2)*;  IV:3329219 to IV:10211735 is JU1580;  IV:2279105 and IV:11083410 are N2. |  |
| JU2195 | *mfIR43(IV,JU1580>N2)*; IV:7607965 to IV:11083410 is JU1580;  IV:7048084 and IV:15829314 are N2. |  |
| JU2196 | *mfIR41(IV,JU1580>N2)*;  IV:3329219 to IV:6676736 is JU1580; IV:2279105 and IV:6699208 are N2. |  |
| JU2197 | *mfIR42(IV,JU1580>N2)*; IV:3329219 to IV:6544139 is JU1580; IV:2279105 and IV:6544139 are N2. |  |
| JU2209 | *mfIR40(IV,JU1580>N2)*;  IV:3329219 to IV:7976415 is JU1580; IV:2279105 and IV:8263671 are N2. |  |
| WM27 | *rde-1(ne219)* |  |
| RB2519 | *drh-1(ok3495)* |  |
| RB1024 | *drh-2(ok951)* |  |
| RB798 | *rrf-1(ok589)* |  |
| NL2098 | *rrf-1(pk1417)* |  |
| SX2408 | *drh-1(ok3495); rde-1(ne219)* |  |
| WM206 | *drh-3(ne4253)* |  |
| SX2409 | *drh-3(ne4253); drh-1(ok3495)* |  |
| WM49 | *rde-4(ne301)* |  |
| SX2407 | *drh-1(ok3495); rde-4(ne301)* |  |
| PD8753 | *+/hT2[qIs48](I;III); dcr-1(ok247)III / hT2[qIs48](I;III)* |  |
| WM160 | *sago-1(tm1195)* |  |
| WM154 | *sago-2(tm894)* |  |
| WM191 | *sago-2(tm894); ppw-1(tm914); ppw-2(tm1120); F55A12.1(tm2686); R06C7.1(tm1414); Y49F6A.1(tm1127); ZK1248.7(tm1113); F58G1.1(tm1019); C16C10.3(tm1200); sago-1(tm1195); T22H9.3(tm1186); R04A9.2(tm1116)* |  |
| SX2375 | *mjIs225[WRM0640dC01;myo-3::gfp::unc-54]* |  |
| SX2376 | *mjIs226[WRM0640dC01;myo-3::gfp::unc-54]* |  |
| SX2377 | *mjIs227[WRM0640dC01;myo-3::gfp::unc-54]* |  |
| GR1373 | *eri-1(mg366)* |  |

**B**

**Table 2: Oligonucleotides used in this study**

| **Name/SNP position/other** | **Primer sequence** | **Comment** |
| --- | --- | --- |
| **I_1817142** | TTCCGGGTACTACAATTAGAGGAG | Forward |
| I_1817142 | CGCCACGTCATATCGATTTTTA | Reverse |
| I_1817142 | TTTTATTATTGATTTTTTGA | Sequencing |
| **I_11290586** | TTGGGAGTCTGATCCAACTGTA | Forward |
| I_11290586 | TGTAGCTGCTGAACTAGACGATGA | Reverse |
| I_11290586 | TCCAAAACAAACCAGTAA | Sequencing |
| I_13811415 | ACCTATATTCCAAGCCATTTGAAA | Forward |
| **I_13811415** | TAACTTTACTCTTTTGGCTGCAAC | Reverse |
| I_13811415 | TGAAATTTAGCATGTGAAC | Sequencing |
| **II_6921473** | AAAAGATTCGGTAGATATGTTCCA | Forward |
| II_6921473 | TCATTCAAATCTTCATCCATAACA | Reverse |
| II_6921473 | TTCATCCATAACAAGCC | Sequencing |
| II_13449599 | TCCCACTTTTTCCTTCAAAAATC | Forward |
| **II_13449599** | TCCACCATGAAAAATGTGTGTT | Reverse |
| II_13449599 | CTTCAAAAATCGCAGA | Sequencing |
| **III_255881** | TTGAGCAAAAATCTGGAAATTTAA | Forward |
| III_255881 | ATCGATAATTTCGACTTTGTTTCA | Reverse |
| III_255881 | TCAAGAATTTTCGGAGA | Sequencing |
| **III_6867359** | AAGGAGCAAGATCATTTCGTAGAA | Forward |
| III_6867359 | CGCAAAGAAAGTAAGTGGATTTGA | Reverse |
| III_6867359 | AAGAAAGTAAGTGGATTTGA | Sequencing |
| III_12217468 | TTATCGGACCTGATGGAGAAA | Forward |
| **III_12217468** | TGGTTTTCCACTTGCATCAGTA | Reverse |
| III_12217468 | TCGGACCTGATGGAG | Sequencing |
| **IV_1860953** | GCACAACTCAGACAGCAACAGACA | Forward |
| IV_1860953 | CCCTCCAACTCGGAAATCCTCT | Reverse |
| IV_1860953 | TCGGAAATCCTCTTAATAT | Sequencing |
| IV_2279105 | GAAGGAATTGCCACAGGGTTATG | Forward |
| **IV_2279105** | GACCTCATCATCCGTCGACTCA | Reverse |
| IV_2279105 | AGGAATTGCCACAGG | Sequencing |
| IV_3329219 | CGGATACTGTGATATGCAATTGTG | Forward |
| **IV_3329219** | CCGCTAGTTTAGTAGAGCAAACG | Reverse |
| IV_3329219 | TAGAGTAGAATTTCATAACC | Sequencing |
| IV_3877431 | GATCCAGTGGCTCATAATCCATAA | Forward |
| **IV_3877431** | TTACCCAAAATACGAGCATTTCA | Reverse |
| IV_3877431 | TGGCTCATAATCCATAAGT | Sequencing |
| **IV_5677640** | TGGTACAGCTCCCATTTCTGAC | Forward |
| IV_5677640 | CATCGAACTCGTCAATCACTATGT | Reverse |
| IV_5677640 | TCGACCAAGGATGGA | Sequencing |
| **IV_5949760** | CATTCCGATTATGGTAATTTCTGA | Forward |
| IV_5949760 | AGCAAAAAAAACTTCCAAAAATTG | Reverse |
| IV_5949760 | ACTTCCAAAAATTGGG | Sequencing |
| IV_6124501 | ACCCTAAAAAAACCCGATAAAATT | Forward |
| **IV_6124501** | ATTCTGCAACAAAAAAAATTAAGC | Reverse |
| IV_6124501 | CAGTAAAGTCTCAACAAATG | Sequencing |
| **IV_6259782** | CGAGAAGAGTTGAAGCAAGTAAAA | Forward |
| IV_6259782 | TTTCAGAATGAGCTTTCGATTTTT | Reverse |
| IV_6259782 | GAATGAGCTTTCGATTT | Sequencing |
| IV_6458494 | ATTGCGTGACACTCATTATCTGC | Forward |
| **IV_6458494** | AGTGCCAGTTGTAAGTGTGTTTTG | Reverse |
| IV_6458494 | ACTCATTATCTGCTTGTTCT | Sequencing |
| IV_6567528 | ATGTTTCAACTGCTACAGATACAA | Forward |
| **IV_6567528** | AAAGAAAGTTTCCCCCAAATG | Reverse |
| IV_6567528 | TGAAGTTCAATTCAAAATAA | Sequencing |
| **IV_6597185** | AACGTGGCTCTAACTTAGTCAGCT | Forward |
| IV_6597185 | GCAAAGGCTGGATTTGTCA | Reverse |
| IV_6597185 | GATTTGTCAACCGACTT | Sequencing |
| **IV_6544139** | GCACTCATTTTTGAGAACTTTAAA | Forward |
| IV_6544139 | TTCAGACACGTATAATAATTCAAG | Reverse |
| IV_6544139 | AAGAATATTGAGATAACTAA | Sequencing |
| **IV_6639829** | GCGAAGGTTATCGATTTTAAAAAG | Forward |
| IV_6639829 | TTTGACATTATTCTGGCGACAC | Reverse |
| IV_6639829 | GGGAAGCGATTTTGT | Sequencing |
| **IV_6648546** | ACCCAGTTCAGATACCACAATTC | Forward |
| IV_6648546 | CGCAGAGAAAACAAATCATTACAG | Reverse |
| IV_6648546 | TTTCAAAAACTGGAA | Sequencing |
| IV_6664407 | CGGCATCGAAACCTCACTAGAG | Forward |
| **IV_6664407** | GCAAGCAAACACGCGAAATG | Reverse |
| IV_6664407 | ACCTCACTAGAGGCAGC | Sequencing |
| **IV_6676736** | ACTTCCCAACTCAAATGCATCT | Forward |
| IV_6676736 | TTGTTTACCCGCCTTACTTGTTTT | Reverse |
| IV_6676736 | TTTATTCTGGCCCGT | Sequencing |
| **IV_6567528** | TGAAACATGTCCCAACTTGTATTT | Forward |
| IV_6567528 | AATGCCATGCGTCTGTACATATA | Reverse |
| IV_6567528 | AAGTTATTCATAGCTT | Sequencing |
| IV_6597185 | TGCGTCTCTCTGATTTTTATGCA | Forward |
| **IV_6597185** | AAGGTTGCCTCGATCATCTTC | Reverse |
| IV_6597185 | ACTCTCTCTCTTGGTGTTT | Sequencing |
| IV_7380467 | GTAACACGGAGTCAAAGCTATTTT | Forward |
| **IV_7380467** | TCAATGAACGTTGAGTTATAAAGG | Reverse |
| IV_7380467 | CACGGAGTCAAAGCTAT | Sequencing |
| **IV_7607965** | CAGATGTTCATCAGCATTACAAA | Forward |
| IV_7607965 | TTTCAGCTTCAGACAGATTATCG | Reverse |
| IV_7607965 | GCTTCAGACAGATTATCG | Sequencing |
| IV_7976415 | TGGAGGCGTAGACATAGAATGA | Forward |
| **IV_7976415** | TTTTCCAAACCCACACACAAC | Reverse |
| IV_7976415 | GATAAATTCATTTGGAACAG | Sequencing |
| IV_8263671 | CGGATCCTTCCAACCAACTGA | Forward |
| **IV_8263671** | GCATGTGGATCTTGCTCAGATGA | Reverse |
| IV_8263671 | GAGAAGCTTCGCATCAC | Sequencing |
| IV_10211735 | CGGATTTGGAACTGTTTATCATG | Forward |
| **IV_10211735** | [Btn]CTCCAGGGGAAAATAAAAAGCA | Reverse |
| IV_10211735 | TGGGTTTTTCCAATTAT | Sequencing |
| IV_11083410 | TCGGAAGTGACTTTAATTTTGAGA | Forward |
| **IV_11083410** | TCAACTCCACATGTGAGTAGTGAT | Reverse |
| IV_11083410 | GCAAAAATGTTGTTCAAA | Sequencing |
| IV_15829314 | CCCAAAAATGAAATTGTCAGG | Forward |
| **IV_15829314** | TGAAACATTATAAACTTGGGAAAA | Reverse |
| IV_15829314 | ATGAATAGAGATAATTTAAG | Sequencing |
| **V_1702142** | TGCCAACTCTTCTAATTTCTTCCA | Forward |
| V_1702142 | ACGGCCAAGCTTTGAAAA | Reverse |
| V_1702142 | AAAATGTTGAGTATCCTCAG | Sequencing |
| V_13707251 | ATTGGCTAGCTGTGAGAATTCATA | Forward |
| **V_13707251** | CCACTTGAGAAATGGAGACTAATC | Reverse |
| V_13707251 | CTGCTGGGCTTGTGA | Sequencing |
| V_20186221 | TTTGTGGAATAAAAATGTCTGAAA | Forward |
| **V_20186221** | AAAATAAGTGACGGGATCATCAA | Reverse |
| V_20186221 | AAAAATGTCTGAAAAATATG | Sequencing |
| **X_2958997** | CGATTCCCAACATTGTTCAGGAC | Forward |
| X_2958997 | CGTGGGGTTCTGCATATTCG | Reverse |
| X_2958997 | CTGCATATTCGCTGAC | Sequencing |
| X_9481467 | GCAATTTTTAGTTTCTGGACAGTT | Forward |
| **X_9481467** | ATTATTTGTTTCCGGTCAAGGTT | Reverse |
| X_9481467 | TCTGGACAGTTACCTACTTA | Sequencing |
| X_15369386 | CGAATTGAATTCCTGGAAAAAA | Forward |
| **X_15369386** | TTTTAACTCATTCTGCCTTGTGTG | Reverse |
| X_15369386 | TATTTGTTTTGTATTTTAGG | Sequencing |
| Universal Forward | [Btn]GTGACGTACTAGCAACGC |  |
| Universal Reverse | [Btn]TAGCAGGATACGACTATC |  |
| oTB17 | ACCACGTGTGGACCCATAAA | Forward RNA2 Orsay virus for qPCR |
| oTB18 | GATCGGTTCGTCTTGGAATG | Reverse RNA2 Orsay virus for qPCR, reverse transcription primer |
| GW194 | ACCTCACAACTGCCATCTACA | Forward RNA1 Orsay virus for qPCR |
| GW195 | GACGCTTCCAAGATTGGTATT | Reverse RNA1 Orsay virus for qPCR, reverse transcritpion primer |
| *eft-2* 2F | CTGCCCGTCGTGTGTTCTAC | Forward *eft-2* for qPCR |
| *eft-2* 2R | TCCTCGAAAACGTGTCCTCTT | Reverse *eft-2* for qPCR, reverse transcritpion primer |
| oTB40 | AGCTTGATCCGCATCTCTCA | Reverse genotyping *drh-1* |
| oTB43 | ACTGGATCGGTGAGAACCTAAA | Forward genotyping *drh-1* |
| *niDf250* deletion genotyping | TTCGCGTGATGCTCAAATAC | Reverse genotyping *drh-1* |
| *niDf250* deletion genotyping | TCAGCTCTTTGTGGGTTTTCT | Forward genotyping *drh-1* |
| WT (only) drh-1 qRT-PCR | GTTCGAAAAACTCGCCTGA | Forward *drh-1*(N2) qPCR |
| WT (only) drh-1 qRT-PCR | TTTGTCAAGAATATTTTTCCATTTC | Reverse *drh-1*(N2) qPCR, |
| *drh-1* qRT-PCR, outside deletion 1 | GGAAGGATCGACGAATTGAA | Forward *drh-1* outside deletions |
| *drh-1* qRT-PCR, outside deletion 1 | CCGTGCATTTTGGAAGATAA | Reverse *drh-1* outside deletions, |
| *drh-1* qRT-PCR, outside deletion 2 | GAGCGATTTTCACTACATTCTCG | Forward *drh-1* outside deletion |
| *drh-1* qRT-PCR, outside deletion 2 | TCGTATACCGCTTGCTCTCC | Reverse *drh-1* outside deletion, |
| *Gapdh* qRT-PCR | TGGAGCCGACTATGTCGTTGAG | Forward Gapdh qRT-PCR |
| *Gapdh* qRT-PCR | GCAGATGGAGCAGAGATGATGAC | Reverse Gapdh qRT-PCR |
| *ok3495* deletion genotyping | TAATGCTTGTTGCTCATCCG | Forward *ok3495* deletion genotyping |
| *ok3495* deletion genotyping | ACACGCAACGCAGTTTTATT | Reverse *ok3495* deletion genotyping |
